# Serological evidence of paracoccidioidomycosis infection in pantanal caimans (*Caiman yacare*)

**DOI:** 10.1007/s11259-026-11148-w

**Published:** 2026-03-18

**Authors:** Aline Myuki Omori, Igor Massahiro de Souza Suguiura, Zilca Maria da Silva Campos, Zoilo Pires de Camargo, Eiko Nakagawa Itano, Mario Augusto Ono

**Affiliations:** 1 Laboratório de Imunologia Animal, Departamento de Imunologia, Parasitologia, e Patologia Geral, Universidade Estadual de Londrina, PR 445 Km 380, Londrina, 86055-900 Paraná Brazil; 2 Ministério da Agricultura, Pecuária e Abastecimento, Embrapa Pantanal, R. 21 de Setembro 1880, Corumbá, 79320-900 MS Brasil; 3 Disciplina de Biologia Celular, Universidade Federal de São Paulo, R. Botucatu, 862, Vila Clementino, São Paulo, 04023-062 SP Brazil; 4 Laboratório de Imunologia Aplicada, Departamento de Imunologia, Parasitologia, e Patologia Geral, Universidade Estadual de Londrina, PR 445 Km 380, Londrina, 86055-900 Paraná Brazil

**Keywords:** Paracoccidioides, Epidemiology, Reptile, Animals, Serology

## Abstract

Paracoccidioidomycosis (PCM) is a systemic mycosis of medical and veterinary importance. Infection by fungi of the genus *Paracoccidioides* has been reported in several animal species but has not yet been described in reptiles. This study aimed to investigate serological evidence of *Paracoccidioides* infection in wild caimans (*Caiman yacare*) from the Brazilian Pantanal, Mato Grosso do Sul, a region endemic for PCM. Serum samples from 50 free-ranging caimans were analyzed by ELISA and immunodiffusion using gp43 and exoantigen, respectively. Anti-gp43 antibodies were detected in 30% of the samples by ELISA, whereas no reactivity was observed by immunodiffusion. No significant differences were found between males and females, indicating similar exposure to the pathogen. To our knowledge, this is the first report of serological evidence of *Paracoccidioides* infection in caimans, expanding the known host range of this pathogen.

## Introduction

Paracoccidioidomycosis (PCM) is a systemic fungal disease common in several Latin American countries that affects humans and animals. It is caused by dimorphic fungi of the genus *Paracoccidioides* (order Onygenales, family Ajellomycetaceae), including *P. brasiliensis* sensu stricto, *P. restrepiensis*, *P. venezuelensis*, *P. americana*, and *P. lutzii* (Venturini et al. 2026). In terrestrial hosts, infection is thought to occur primarily through inhalation of fungal propagules from soil (Venturini et al. 2026).

Paracoccidioidomycosis encompasses both symptomatic disease (PCM disease) and asymptomatic infection (PCM infection). PCM disease is rare in animals, with natural cases described in domestic dogs, monkeys, in a cat, and in a southern two-toed sloth (*Choloepus didactylus*) (Johnson and Lang 1977; Gonzalez et al. 2010; Trejo-Chávez et al. 2011; Suguiura et al. 2024). In these cases, clinical signs were generally associated with fungal invasion of the mononuclear phagocyte system, resulting in marked involvement of lymph nodes and spleen; however, dissemination to other tissues and organs has also been reported (Suguiura et al. 2024). Similar manifestations are frequently observed in acute PCM in humans, whereas the chronic form, the most prevalent presentation in this species, is predominantly characterized by pulmonary involvement (Venturini et al. 2026). In contrast, PCM infection is considerably more common and has been documented in a wide range of domestic and wild species, including dogs, cats, livestock, poultry, monkeys, small rodents, armadillos, and fish (Corte et al. 2007; Silveira et al. 2008; Oliveira et al. 2011; Venturini et al. 2026; Suguiura et al. 2019).

Although soil is considered the primary ecological niche of *Paracoccidioides*, evidence of infection has also been demonstrated in an exclusively aquatic species, Nile tilapia (*Oreochromis niloticus*) (Suguiura et al. 2019). These findings are consistent with the hypothesis proposed by Conti-Díaz in 1980, which suggested that the epidemiological cycle of *Paracoccidioides* would involve a wide range of vertebrates and invertebrates, including fish, birds, amphibians, reptiles, and arthropods (Conti-Díaz and Rilla 1989). Despite extensive documentation of infection in mammals and limited reports in birds and fish, infection in reptiles has not been previously reported.

The pantanal caiman (*Caiman yacare*) is a semi-aquatic reptile found in the lowlands of Bolivia, western Brazil, and northern Argentina (Campos et al. 2010). The Pantanal is a subtropical Brazilian biome characterized by a vast sedimentary floodplain, seasonal flooding, and average annual rainfall of 800–1200 mm, conditions that make it a suitable habitat for *Paracoccidioides* (Alho 2009). In addition, the western region of Brazil, which includes part of the Pantanal biome, is a well-known endemic area for both human and animal PCM. Given the lack of data regarding PCM in reptiles, this study aimed to evaluate *Paracoccidioides* infection in caimans from this endemic area.

## Materials and methods

### Serum samples

**Fig. 1:**
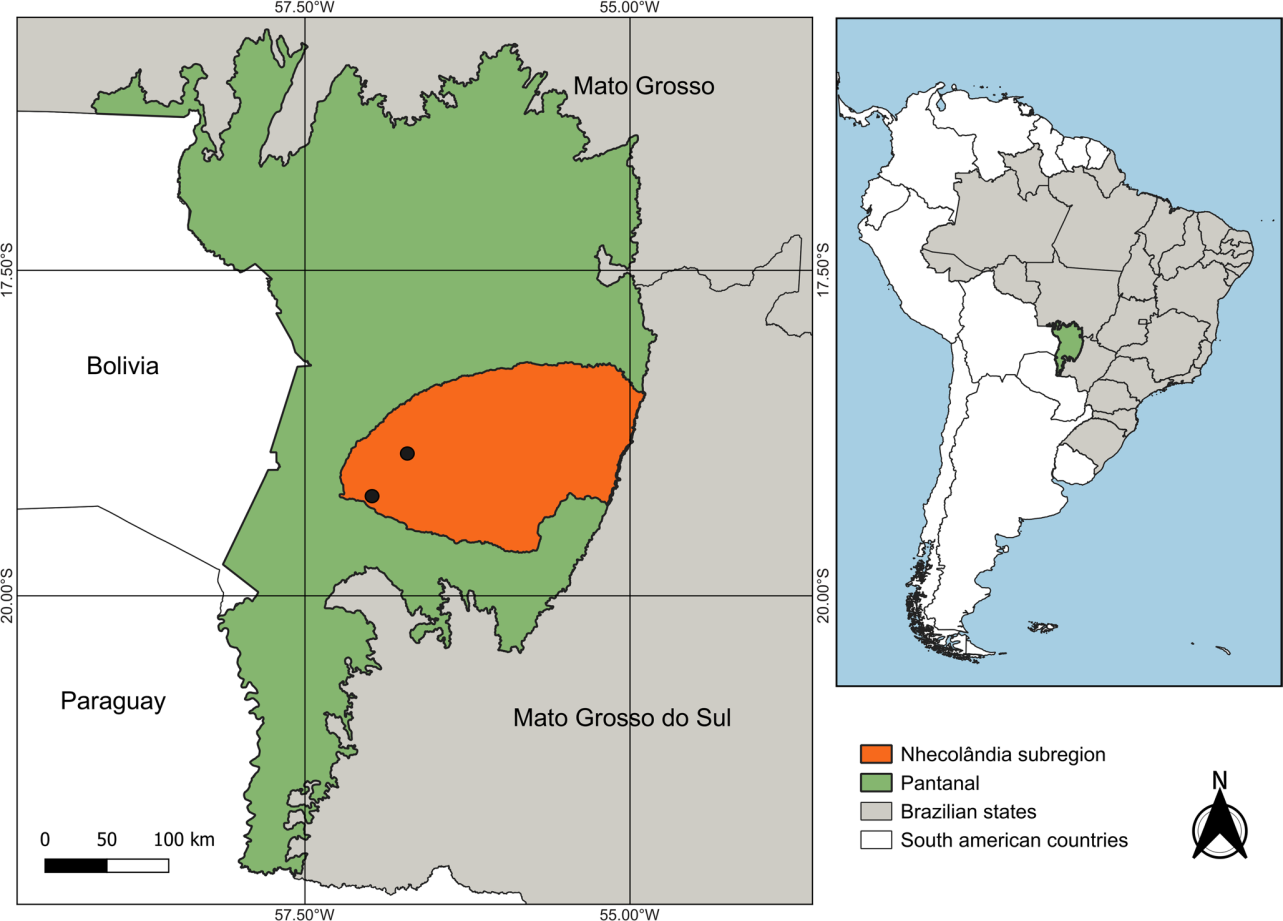
Pantanal region where the caiman samples were collected. Black dots indicate the locations of cattle ranches within the Nhecolândia subregion. Maps were generated using QGIS software version 3.40 (Geographic Information System, Open Source Geospatial Foundation Project; available at https://qgis.org/), with geospatial data provided by IBGE (Brazilian Institute of Geography and Statistics) and Embrapa (Brazilian Agricultural Research Corporation)

A total of 50 apparently healthy adult caimans (*Caiman yacare*) were captured at two cattle ranches in the Nhecolândia sub-region of the Pantanal, Mato Grosso do Sul, Brazil (Fig. 1). Blood samples were collected via internal jugular vein puncture. All procedures were approved by the Brazilian Institute of Environment and Renewable Natural Resources (Protocol No. 18363-1/2009) and the Fiocruz Animal Ethics Committee (CEUA-Fiocruz LW-1/12, Protocol P-74/10 − 5).

### *Paracoccidioides* antigens

The exoantigen was obtained from culture of *P. brasiliensis* B339 as described by Camargo et al., and the gp43 antigen was purified from *P. brasiliensis* B339 exoantigen by immunoaffinity chromatography as described by Puccia and Travassos (Camargo et al. 1988; Puccia and Travassos 1991).

### Indirect ELISA

The caiman serum samples were analyzed by indirect ELISA using purified gp43 as antigen. The flat bottom microtiter polystyrene plates were coated with gp43 (250 ng/well) in carbonate bicarbonate buffer 0.2 M pH 9.6 at 4 °C overnight. After washing with PBS-T, the plate was blocked with 5% skim milk in PBS for 1 h. After washing with PBS-T, the caiman serum samples (1:50) in 1% skim milk PBS was added and incubated for 1 h. The wells were washed with PBS-T and incubated with chicken IgY-anti caiman immunoglobulin for 1 h. After washing with PBS-T, anti-chicken IgY-peroxidase (Cat. No. A16054, Invitrogen) in 1% skim milk PBS was added and incubated for 1 h. The plate was washed with PBS-T and incubated with substrate-chromogen solution (H_2_O_2_/TMB) and the reaction was stopped with 4 N H_2_SO_4_. The absorbance was measured at 450 nm. Serum samples with two and half times the absorbance of the well without caiman serum were considered positive (Corte et al. 2007).

### Double immunodiffusion test

The caiman serum samples that were positive in indirect ELISA were analyzed by double immunodiffusion (DID) test as described by Camargo et al. (Camargo et al. 1988).

### Statistical analysis

The data were analyzed by the Fisher exact test using BioEstat 5.3 program. The differences were considered statistically significant when *p* < 0.05.

## Results

Caiman serum samples showed reactivity to the *P. brasiliensis* gp43 antigen in the indirect ELISA, revealing an overall positivity rate of 30% (15/50) across the sampled population. When analyzed by sex, the prevalence of antibodies was relatively similar between groups: 31% of females (5/16) and 29% of males (10/34) tested positive for the antigen and this difference was not statistically significant (*p* > 0.05). Notably, all samples that tested positive in the ELISA yielded negative results when further evaluated by double immunodiffusion (DID).

## Discussion

The order Onygenales includes several well-established reptile pathogens, particularly emerging fungi from the genera *Ophidiomyces*, *Nannizziopsis*, and *Paranannizziopsis* (family Onygenaceae) (Paré and Sigler 2016). These taxa, previously classified as the “*Chrysosporium* anamorph of *Nannizziopsis vriesii*” (CANV), are now recognized as significant threats to reptile populations worldwide (Paré and Sigler 2016). In contrast, members of the family Ajellomycetaceae, also within Onygenales, are classically associated with systemic infections in warm-blooded animals. Nevertheless, our findings provide evidence supporting the potential susceptibility of reptiles to *Paracoccidioides* spp., as demonstrated by the detection of antibodies against the principal antigen of this genus in free-ranging Pantanal caimans.

ELISA results revealed a 30% seropositivity rate for anti-gp43 antibodies in the sampled caimans (Table 1). No significant differences were observed regarding sex (*p* > 0.05), suggesting that males and females are equally exposed to the pathogen, as also observed in other animal species (Corte et al. 2007; Oliveira et al. 2011). Remarkable sex-related differences in prevalence are observed only in humans with chronic PCM, in whom the disease is more common in men than in women (Venturini et al. 2026). This disparity is attributed to the inhibitory effect of estrogen on the conversion of the infective mycelial form to the pathogenic yeast form (Shankar et al. 2011). Because the clinical manifestation of PCM in animals more closely resembles the acute form of the disease in humans, sex-related differences appear to have little influence on the occurrence of the disease in animals.

**Table 1:** Reactivity of the caiman serum samples to *P. brasiliensis* gp43 antigen evaluated by indirect ELISA according to sex

Gender	Positive n (%)	Negative n (%)	Total
	ELISA	ELISA	
Female	5 (31)	11 (69) 24 (71) 35 (70)	16 (32)
Male	10 (29)		34 (68)
Total	15 (30)		50 (100)

ELISA is a highly sensitive method for detecting PCM infection and can identify antibodies against all cultivable *Paracoccidioides* species, even when crude antigen preparations with heterogeneous composition are used (Suguiura et al. 2025). The 43 kDa glycoprotein (gp43) is the main antigen employed in PCM serological diagnosis, including ELISA, and it is expressed in both the mycelial and yeast phases of the fungus (Travassos et al. 2004). This expression in both morphological forms makes gp43 a suitable marker for detecting infection in ectothermic hosts, such as caimans, in which the pathogenesis of PCM and the infective fungal form remain unknown.

In endothermic animals, fungal propagules convert into the pathogenic yeast form following inhalation; however, ectotherms lack the high body temperature typically required to trigger this dimorphic conversion. Despite the absence of this thermal stimulus, the fungus is still able to enter the organism and elicit a humoral response, as evidenced by the detection of fungal DNA in tissues and circulating antibodies in fish (Suguiura et al. 2019). Interestingly, experimental studies using invertebrate models kept at 25 °C did not observe a conversion from yeast to mycelia after three days of incubation (Thomaz et al. 2013). In contrast, standard laboratory cultures at room temperature typically show transitional yeasts with elongated buds within 36 h and visible hyphae formation by 60 h (Ramírez Martínez 1971).

Regarding the negative results in the DID, it is important to note that while this test is a standard diagnostic method for humans, it can fail in approximately 10% of patients with microbiologically proven disease (Do Valle et al. 2001). In veterinary medicine, negative DID results have also been documented in animals with PCM disease (Suguiura et al. 2024). These false negatives can occur for several reasons, including the presence of asymmetric antibodies, and antigenic differences between the laboratory-prepared antigens and the specific species of *Paracoccidioides* infecting the host. In caimans, the correlation or co-participation of these factors remains unknown, especially when considering the potential differences in immunological responses to *Paracoccidioides* antigens in this species.

Despite extensive research, the eco-epidemiology of PCM is not yet fully understood. While terrestrial animals are traditionally considered more at risk due to soil contact, semi-aquatic reptiles like *C. yacare* are likely exposed in both environments. Caimans maintain close contact with the soil during nesting, egg incubation, and basking along riverbanks; however, they may also be exposed to the pathogen within the aquatic environment.

Given that *Paracoccidioides* spp. grow and produce infectious conidia in highly humid soils, and that the Pantanal is characterized by high temperatures, humidity, and seasonal flooding, this wetland environment is likely favorable for the development of the fungus (Alho 2009; Terçarioli et al. 2007). The presence of antibodies against *Paracoccidioides* has been detected in other species within this biome. Our group previously reported high rates of *Paracoccidioides* infection in dairy cattle and chickens from the Pantanal, further supporting the widespread occurrence of the pathogen in this region (Silveira et al. 2008; Oliveira et al. 2011).

In brief, this study provides the first serological evidence of *Paracoccidioides* infection in caimans, indicating that PCM affects a broader range of animal species than previously recognized. Further studies are needed to investigate interactions between ectothermic hosts and these fungi, including improved approaches for fungal detection and identification, as well as the assessment of host responses to infection. Such efforts are essential to clarify the ecological role and distribution of *Paracoccidioides* in wildlife and to advance understanding of host–pathogen dynamics across diverse ecosystems.

## Data Availability

No datasets were generated or analysed during the current study.
